# Adulteration of Brain Health (Cognitive, Mood, and Sleep Enhancement) Food Supplements by the Addition of Pharmaceutical Drugs: A Comprehensive Review of Analytical Approaches and Trends

**DOI:** 10.3390/foods13060908

**Published:** 2024-03-16

**Authors:** Rafael Paiva, Manuela Correia, Cristina Delerue-Matos, Joana S. Amaral

**Affiliations:** 1 REQUIMTE/LAQV, Instituto Superior de Engenharia do Porto, Instituto Politécnico do Porto, Rua Dr. António Bernardino de Almeida 431, 4249-015 Porto, Portugal; rafaelpaiva_t5@hotmail.com (R.P.); cmm@isep.ipp.pt (C.D.-M.); 2 Centro de Investigação de Montanha (CIMO), Instituto Politécnico de Bragança, Campus de Santa Apolónia, 5300-253 Bragança, Portugal; 3 Laboratório Associado para a Sustentabilidade e Tecnologia em Regiões de Montanha (SusTEC), Instituto Politécnico de Bragança, Campus de Santa Apolónia, 5300-253 Bragança, Portugal

**Keywords:** authenticity, dietary supplements, plant food supplements, food safety, pharmaceutical drugs, analogues

## Abstract

In recent years, the consumption of dietary supplements has grown worldwide, particularly in developed regions. However, this growing market has also become a prime target for adulteration practices, with some manufacturers illegally adding pharmaceuticals into plant-based food supplements (PFS) to enhance their effects. While extensive research has focused on detecting adulterant drugs in PFS tailored for improving sexual performance, weight loss, and muscle building, less attention has been given to supplements intended for mood enhancement, sleep aid, and cognitive function (nootropics). Nonetheless, recent reports indicate an increasing level of adulteration within this group of PFS. Therefore, this review aims at providing a comprehensive overview on the adulteration of PFS tailored for brain health, with a focus on the analytical techniques utilized for detection while also presenting data on consumption patterns and the prevalence of reported adulterants. Considering that the detection of such fraudulent practices primarily relies on chromatographic techniques coupled with mass spectrometry (MS), the developments in this field comprising either targeted or untargeted analysis of pharmaceutical adulterants are discussed.

## 1. Introduction

Food supplements, also known as dietary supplements, are products for oral consumption in controlled dosages, such as pills, capsules, tablets, ampoules, liquids, and powders, which are intended to complement the regular diet and support overall health. Frequently, these products include ingredients such as vitamins, minerals, amino acids, herbal products, and enzymes, as well as other substances, such as excipients. Many food supplements are commonly designated as plant food supplements (PFS), as they contain one or various botanical ingredients known to be biologically active in humans, with many of these plants also being traditionally used in herbal medicine practices [1]. According to several reports, PFS are increasingly used by the global population, with several factors explaining this increase, such as a growing trend for natural and holistic approaches to maintain health and promote overall well-being, PFS are sometimes more affordable than pharmaceuticals, they are easily obtained in supermarkets, herbal shops, or on websites, and they are heavily advertised as natural products, which frequently misleads consumers to perceive them as being safe and without possible side effects [2,3,4]. Moreover, different studies refer to a growing mistrust in conventional therapies and pharmaceutical drugs, leading to an increasing number of persons resorting to alternative medicines and self-care for their long-term health issues [5,6]. Finally, since PFS are dispensed in forms visually identical to pharmaceuticals, consumers erroneously believe that they have also been approved for safety and efficacy by control agencies before marketing and are also produced following the same quality control standards applied in the pharmaceutical industry [7].

In addition to the undeniable growth of the food supplements market, both in supply and demand, the global market for these products is projected to continue to grow. According to Statista, a data analysis company, the global market of these products was estimated at around 137 billion USD in 2021, presenting a forecasted compound annual growth rate of around 9% until 2028 [8], probably reflecting an increase in the demand for immunity-boosting products since the COVID-19 pandemic. Being highly consumed products and representing a high-value market, in recent years, numerous reports have been exposing these products as growing targets for economically motivated adulterations. In general terms, the adulteration of food supplements consists of a deviation between what is written on the product’s label and its content [3]. There are several ways in which manufacturers can adulterate food supplements, such as by adding one or more substances that are not indicated in the label, by wrongfully specifying the concentration of a compound/ingredient, by using novel non-authorized ingredients, and in the case of PFS, by using plant(s) of lower quality or a different plant part (e.g., the stems instead of the flowers, etc.), or even by using different plant species that are added as fillers [2]. Some manufacturers may resort to these tactics to overcome shortages of botanical products and/or to increase the profit margin [3]. In fact, botanical adulterations are one of the most common types of fraud in PFS. Recently, different papers have reviewed different approaches and methodologies for detecting botanical frauds in PFS and may be consulted for detailed information on this subject [9,10]. Another type of adulteration frequently reported in food supplements concerns the addition of active pharmaceutical ingredients (APIs) to ensure the effects advertised for the product, misleading consumers into believing that the efficacy was due to the properties of the plant ingredients [11,12]. Food supplements adulterated with APIs are a major public health problem since they may seriously affect the health of consumers, with several cases of adverse effects, hospitalizations, and even deaths being reported [13,14]. Moreover, the presence of slightly modified molecules (API analogues) to circumvent their detection by the authorities has also been detected in these products, which further increases the associated risk since for most of these compounds, no studies exist about their safety, toxicity, and efficacy [15,16].

### 1.1. Food Supplements Regulation

#### 1.1.1. Food Supplements Legislation

Legislation differs from country to country and can be undermined by the blurred definitions and the differences in the scope, requirements, and terminology, particularly in what concerns food supplements and herbal medicines categories [6]. In the EU, food supplements are considered foods and, therefore, must respect the principles and requirements of the food law (Regulation (EC) No. 178/2002) [17], such as conforming with hygiene regulations (EU directive 852/2004) [18] and with the limit levels of contaminants under the Commission Regulation (EU) 2023/915 [19]. Moreover, food supplements are specifically defined in Directive 2002/46/EC as “…foodstuffs the purpose of which is to supplement the normal diet and which are concentrated sources of nutrients or other substances with a nutritional or physiological effect, alone or in combination, marketed in dose form, namely forms such as capsules, pastilles, tablets, pills, and other similar forms, sachets of powder, ampoules of liquids, drop dispensing bottles, and other similar forms of liquids and powders designed to be taken in measured small quantities” [20]. This directive includes two annexes, one that lists the vitamins and minerals that may be used in the manufacturing of food supplements, and another that declares specific substances allowed to be used as vitamins and minerals (e.g., amino acids, enzymes, essential fatty acids, prebiotics, and probiotics). In addition, Regulation (EC) No. 1925/2006, on the addition of vitamins and minerals and of certain other substances to foods, should also be considered. Besides regulating the vitamins, minerals, and corresponding sources, this regulation and further amendments maintain a list of prohibited and restricted substances, which are known or suspected to have adverse effects on health and the use of which is, therefore, controlled (Annex III of Regulation (EC) No. 1925/2006) [6,21]. In another category (classified as medicines), herbal medicinal products are regulated in the EU as medicines by Directive 2004/24/EC, which determines they can only be commercialized after safety and efficacy validation by the European Medicines Agency (EMA) [22]. In the U.S., these products are known as dietary supplements and are considered a special category of foods being regulated by the Dietary Supplement Health and Education Act (DSHEA) of 1994, which defines them as those products other than tobacco that are intended to supplement the diet and that contain one or more of the following dietary ingredients: vitamins, minerals, herbs, or other botanicals; amino acids; dietary substances used by man to supplement a diet by increasing the total dietary intake; concentrates, metabolites, constituents, extracts, or a combination of the mentioned ingredients, intended to be taken by mouth as a pill, capsule, tablet, or liquid [23]. In the U.S., the Food and Drug Administration (FDA) regulates both finished dietary supplement products and dietary ingredients, but contrary to drugs that need to prove their safety and efficacy before being approved, dietary supplements do not require an approval or assessment by the FDA prior their introduction into the market. Nevertheless, new dietary ingredients introduced after 1994 should undergo a safety review through the New Dietary Ingredient Notification (NDIN) process, although with some exceptions [6]. Overall, the FDA is minimally involved in the regulation of this industry, with manufacturers being the ones responsible for the commercialization of safe, effective, and non-adulterated dietary supplements [5] and products being mainly regulated through post-market surveillance [6]. In other regions of the globe, such as in South Korea, dietary supplements are overseen by the Ministry of Food and Drug Safety (MFDS) and must respect the “Food Sanitation Act” and “Custom Law”. In this case, all ingredients present in the products must have individual market authorization by the Korean Food and Drug Administration [5]. Additional information about legislation and the status of dietary/food supplements and herbal medicines in other countries, such as Canada, Australia, New Zealand, China, and Japan, can be consulted in the review paper on the regulatory landscape of dietary supplements and herbal medicines published after the Global Summit on Regulatory Sciences that took place in Beijing in 2018 [6]. Further details on the regulatory guidelines of dietary supplements in the U.S. can also be consulted in the review by Bailey [24].

#### 1.1.2. Food Supplements Regulation by the FDA in the U.S.

In recent years, the FDA has been strengthening its efforts regarding the regulation of dietary supplements and the broadening of public awareness. In this sense, in 2019 the FDA implemented a “Dietary Supplement Ingredient Advisory List”, which was updated to the “Dietary Supplement Ingredient Directory” by the end of 2023. Additionally, the FDA has been releasing several public notifications advising consumers not to purchase or use products for which hidden drug ingredients were identified, as well as issuing warning letters to firms whose products are not compliant with legislation, which frequently results in voluntary recall or market withdrawal of the products. The “Dietary Supplement Ingredient Directory” does not intend to be a comprehensive list of all ingredients used in products marketed as dietary supplements; instead, it has the overall aim of helping manufacturers, retailers, and consumers to stay informed. For that purpose, it includes a list of particular ingredients together with respective links to the FDA’s actions and communications [25]. Moreover, the FDA also keeps a “Health Fraud Product Database” listing unapproved products subjected to health fraud-related violations, in which supplements presenting undeclared ingredients or new dietary ingredients are included [26]. Searching this database from 2007 until December 2023, a total of 1967 products were listed, of which 1264 (64.2%) concerned the presence of undeclared APIs. In line with data previously reported [11], from the supplements tainted with pharmaceuticals, the most frequent were products for sexual enhancement (53.6%), which are frequently adulterated with phosphodiesterase-5 (PDE-5) inhibitors or, less often, with a mixture of PDE-5 inhibitors and antidepressants. This was followed by weight-loss products (34.5%), mainly adulterated with banned anorexics, such as sibutramine, but also with antidepressants (mainly fluoxetine), stimulants, PDE-5 inhibitors, anxiolytics, diuretics, and anti-inflammatory drugs. Supplements for bodybuilding (8.6%) mainly contain anabolic steroids, supplements for joint pain, arthritis, and/or gout (2.5%) contain anti-inflammatory APIs, and finally others (0.9%), which are sold for different purposes, such as brain function, lowering cholesterol, and stimulating appetite. Among this last group, recently, in November 2023, the FDA issued a warning to consumers against products containing tianeptine, a non-FDA-approved drug that claims to improve brain function and treat anxiety and depression. Several consumers reported seizures and loss of conscience after consuming these supplements, leading to several hospitalizations [27]. Previously, in 2019, public notifications were issued for two supplements sold for sleep improvement, which were revealed to contain the sedative-hypnotics eszopiclone and zopiclone.

#### 1.1.3. Notifications in the EU Rapid Alert System for Food and Feed (RASFF)

In the EU, much of the knowledge on the prevalence of adulteration of dietary supplements comes from the Rapid Alert System for Food and Feed (RASFF). This food alert system covers a wide range of products, including food supplements, and provides a framework for the swift communication and rapid sharing of information on safety issues among member countries, playing a crucial role in assuring the safety of food products in the European market. Recently, Amidžić et al. [28] used the data available on RASFF to review the notifications of pharmaceuticals illegally added to food supplements. To this aim, the authors conducted a search within the database from 2011 to 2022, specifically targeting the product category “dietetic food, food supplement, and fortified food” linked to the hazard category “composition”. The automatic search retrieved a total of 1206 records, with 982 on food supplements, of which 474 (48.3%) were identified as cases of adulteration with pharmaceuticals. Similar to the U.S., several products were found to be adulterated with multiple substances, with the most frequent being PDE-5 inhibitors (37%), stimulants (34%), and anorexics and laxatives (14%), with lower numbers being found for cannabinoids (5%), nootropics (4%), anabolic androgenic steroids (2%), and other drugs, such as antibiotics and hypnotics (4%) [28]. Conducting a new search using RASFF data from January 2020 to December 2023, without restricting the hazard category to “composition” but instead encompassing all hazard categories, we retrieved a total of 1290 records that were manually refined for the presence of API or non-authorized substances. Due to the differences in the selection of the “hazard category”, our outcome was slightly different from that reported by Amidžić et al. [28]. Namely, besides retrieving notifications due to additional substances, such as L-alpha-glycerylphosphorylcholine (α-GPC), which was not considered in the study of Amidžić et al. [28] because it falls under the hazard category “novel foods” instead of “composition”, for other substances we obtained a higher number of notifications (as they were present in more than one category). For instance, Amidžić et al. [28] reported a total of 50 notifications due to the presence of cannabinoids (23 for delta-9-tetrahydrocannabinol (THC), 14 for cannabidiol (CBD), and 13 for THC+CBD) for the period 2011–2022, while in the shorter period of 2020–2023 we retrieved 185 notifications related to cannabinoids (122 due to CBD, 44 due to THC, and 19 due to CBD+THC or cannabigerol), as in the last years they seem to be mainly classified under the hazard category “novel foods”.

Of the manually refined returns, 249 were identified as having one or more unauthorized substances, while 17 were generally described as containing undeclared prescription ingredients, pharmacologically active ingredients, unauthorized or prohibited substances, and prohibited ingredients, without specifying the substances. Similar to the U.S., the most common was the addition of one or more PDE-5 inhibitors (50 products) or their analogues (2 products). In the food supplements marketed for sexual potency, we also included those notified in RASFF due to the presence of yohimbine (*n* = 45), an unauthorized substance in supplements marketed in the EU. Despite being an alkaloid from *Pausinystalia johimbe* bark, which is traditionally used as an aphrodisiac, yohimbine chloride is considered a prescription drug in the U.S., although it has been mostly replaced by PDE-5 inhibitors due to its limited efficacy and significant adverse effects [29]. Recently, yohimbine has also been promoted among bodybuilders as a fat burner, which possibly explains its presence together with stimulants in 10 additional products. In the EU, the presence of stimulants was also revealed to be high, mainly due to the alkylamine 1,3-dimethylamylamine (DMAA), but also to 1,3-dimethylbutylamine (DMBA), 2-amino-6-methylheptane (DMHA), ephedrine, 2-phenylethylamine, octopamine, and synephrine, resulting in 31 notifications. Unlike the U.S., the prevalence of the anorexic sibutramine was low, corresponding only to 12 products. Notably, 2,4-dinitrophenol, an anorexic with significant acute toxicity, led to eight notifications, contrasting with its absence in the FDA’s Health Fraud Product Database search. The number of products related to bodybuilding or workout supplements notified by RASFF (*n* = 11; 4.4%) was also lower, as compared to those in the U.S., due to either the presence of anabolic androgenic steroids or to selective androgen receptor modulators (SARMs). Interestingly, when compared to data retrieved from the FDA database, a substantially higher number of notifications in the EU pertained to substances associated with brain function. In this group, we considered not only APIs, but also unauthorized substances considered as novel foods, such as α-GPC and 5-hydroxytryptophan (5-HTP). Among those notifications, two concerned the presence of pharmaceutical drugs such as lithium, a psychiatric drug employed as a mood stabilizer in conditions such as mania and bipolar disorder, and vinpocetine, a drug sold in the EU under the brand name Cavinton for the symptomatic treatment of cognitive changes or as a supportive treatment in acute ischemic stroke since it can improve brain blood flow [30]. Previously, the presence of α-methylphenylethylamine (brand name Adderall) used to treat neurological disorders, such as attention deficit and hyperactivity disorder (ADHD), has been notified in RASFF [28].

Besides pharmaceutical drugs, between 2020 and 2023, other substances with biological activity at the brain level triggered RASFF notifications. Noteworthy, among these was α-GPC, notified in 25 products. α-GPC, a precursor of acetylcholine assumed to enhance cognitive function and support memory and learning, is one of the most used cholinergic compounds due to its ability to cross the blood–brain barrier [31]. While α-GPC is found as an ingredient in dietary supplements in the U.S. and is a recently approved ingredient by Health Canada’s Food Directorate for use in supplemented foods [32], it is considered a novel food in the EU since it was not consumed to a significant degree as a food before 15 May 1997, therefore requiring a pre-market authorization [33]. Moreover, discrepancies in the use of this substance between countries have been reported since it is considered a prescription drug in some EU countries [34], such as in Italy where it is sold under the brand name Delacit.

As a natural substance extracted from *Griffonia simplicifolia* seeds, 5-HTP is another ingredient frequently used in dietary supplements for brain activity sold in the U.S., in this case for improving mood and sleep. 5-HTP can be synthesized in the body from the essential amino acid tryptophan, being the immediate precursor of serotonin, a neurotransmitter that plays a crucial role in regulating mood, reward, and cognition, among other physiological processes. Moreover, 5-HTP can also be transformed into melatonin, a hormone that regulates the sleep–wake cycle [35]. While in the U.S. it is not used as a prescription drug, in different EU countries this molecule is an authorized medicine for the prophylactic therapy of migraine and post-anoxic myoclonic encephalopathy (Lance-Adams syndrome) [36]. The nutrition and health claim of enhancing mood and attention, supporting its use in food supplements, was negatively evaluated by EFSA under Regulation (CE) 1924/2006 [37]. Currently, 5-HTP is considered a novel food in the EU, thus leading to 25 notifications between 2020 and 2023. Melatonin is another substance that can be either used as a food supplement or a medicine in the EU. In 2011, a nutrition and health claim related to melatonin and the reduction of sleep onset latency was evaluated by EFSA, which concluded that the claimed effect can be obtained by consuming 1 mg of melatonin close to bedtime [38]. Therefore, several EU countries currently authorize melatonin as an ingredient in food supplements up to 1 mg per dose and per day. Nevertheless, the regulatory status of melatonin varies among countries, with Latvia and France allowing up to 2 mg, others such as Germany considering products containing 0.28 mg of melatonin or more per dose and per day as being medicines, and others such as Denmark, the Czech Republic, and Slovenia not authorizing its presence in food supplements [39]. Therefore, a total of seven notifications concerning melatonin were issued between 2020 and 2023 in RASFF.

Gamma-amino butyric acid (GABA) is another substance extensively used in the U.S. as an ingredient in dietary supplements marketed for the relief of anxiety and to elevate mood [40]. While it is allowed in the U.S. and several EU countries, two notifications were issued: one due to unauthorized claims and another because GABA is classified as a medicinal substance in Finland by the Finnish Medicines Agency.

Finally, two other substances exhibiting activity at the brain level, namely huperzine A and dimethylaminoethanol (DMAE), have been notified in RASFF for their presence in food supplements. Huperzine A is an alkaloid from *Huperzia serrata*, a traditional Chinese medicine that showed strong anticholinesterase activity in pharmacological studies [41]. Currently, huperzine A can be found in many dietary supplements sold in the U.S. that are marketed with claims of enhancing brain health, memory, concentration, and focus. Despite being a natural compound, the amounts frequently included in dietary supplements sold in the U.S. are regularly too high to have a natural origin. In the EU, regardless of the source, huperzine A is considered an unauthorized ingredient in food supplements, being responsible for 27 notifications between 2020 and 2023. Similarly, DMAE, a hydroxy derivative of choline with the capacity to modulate the cholinergic system, is also accepted as an ingredient in dietary supplements sold in the U.S. due to its natural presence in fish, such as salmon, mackerel, and sardines, while being considered an unauthorized substance in Europe. In fact, different DMAE salts have been used in pharmacotherapy both in European countries and the U.S. for the treatment of disorders of the central nervous system, such as dementia in the geriatric population associated with the hypofunction of cholinergic neurons, treatment of behavioral problems, ADHD, or as agents to support memory, focus, and improved learning. Being an unauthorized substance in food supplements in the EU, it was responsible for six notifications in RASFF in the last four years.

Overall, the information compiled from RASFF and the FDA Health Fraud Product Database clearly demonstrates that several food supplements highly demanded by consumers are targets of adulteration by the illegal addition of pharmacologically active substances. Nevertheless, the estimated number of fraud cases is most probably under-represented due to the low number of controls/analyses by governmental agencies and unreported cases by consumers that experienced secondary effects. This hypothesis is supported by different papers that reported the identification of molecules, other than the ones mentioned, in food supplements marketed for improving cognition, sleep, or mood, such as piracetam, phenibut, and picamilon [42,43], adrafinil (an analogue of modafinil) [44], diazepam, alprazolam, and clonazepam [12], and phenobarbital [45], among others.

### 1.2. Food Supplements’ Consumption

The consumption of food supplements has increased dramatically in the last decades, particularly in developed countries and in economically advanced economies, such as in the North American region, the EU, and in the Asia-Pacific region [5]. Presently, it is estimated that up to 80% of the world’s population uses food supplements or herbal medicines regularly [6]. Food supplements can present a wide range of different formulations, as they are consumed for different purposes and outcomes, including the improvement of health and lifestyle, preventing diseases by maintaining good health, or the enhancement of physical or mental performance. Moreover, some of the available supplements come in a spectrum of sub-products tailored for different groups of the population, such as children, particular groups of adults (athletes, menopausal women, etc.), and the elderly. Understanding the global and regional usage of food supplements, and particularly of PFS, has been limited by the lack of specific literature and the fact that most published studies have focused on the U.S. and, to a much lesser extent, on some European countries and other regions. Moreover, there are few studies specifically conducted on the use of PFS in certain age and/or conditional groups (e.g., pregnant women, immunocompromised patients, students, etc.), and most available data come from large surveys of government health agencies, such as the USA National Health and Nutrition Examination Surveys (NHANES) and the National Health Interview Surveys (NHIS) [5,6]. Data from these surveys demonstrate that the consumption of supplements in the U.S. increased steadily until 2000, after which the use of these products slowed down until 2012, when about 52% of the population was using them regularly [5]. A recent study that analyzed data from NHANES 1999–2018, regarding adults with diabetes, reported that 54.0% of the 8122 adults included in the study used supplements regularly, of which the most popular were vitamins (87.3%), minerals (75.3%), and botanicals (51.8%) [46]. The work of Garcia-Alvarez and colleagues, regarding the European multi-country (Finland, Germany, Italy, Romania, Spain, and the United Kingdom) PlantLIBRA consumer survey, revealed a lower value, since only 18.8% of the screened respondents from a sample of 2359 adult population used at least one plant-based food supplement [47]. Later, the prevalence of PFS consumption in subgroups of different ages and genders in the Netherlands was reported by Jeurissen et al. [48]. The authors considered the data from the Dutch National Food Consumption Surveys (DNFCSs) in 2007–2010 and 2010–2012, and a specific PFS consumption survey using online questionnaires conducted in 2014, in two phases: first, a screening survey using a representative sample of 75,100 adults and children; then, 739 PFS users were selected for a more detailed survey. Overall, the authors reported an increased prevalence of PFS usage in the Dutch population in 2014, as compared with the 2007–2012 DNFCFs surveys, highlighting the usage of around 600 different PFS, comprising 345 different botanicals, with the most frequent being echinacea (Echinacea purpurea), ginkgo (Ginkgo biloba), cranberry (Vaccinium macrocarpon), and ginseng (Panax ginseng). Interestingly, this study, which was the first to provide data on PFS consumption among children in Europe, evidenced differences between the PFS types used among different subgroups. Despite that the “improvement of the immune system/defense system” was the main reason for the use of PFS in children (31%), men (17%), and women (19%), the following motives were different among the three subgroups, namely, flu/cold (12%), energy (9%), relaxing (6%), and general health (6%) for children, energy (11%), heart/blood circulation (7%), general health (6%), and digestive function (6%) for men, and urinary tract (8%), energy (8%), digestive function (7%), and other (7%) for women. More recently, a cross-sectional study based on in-person questionnaires collected from 2018 to 2019 among the Greek population, with a final sample size of 28,491 respondents aged more than 15 years old, demonstrated that 55.5% used dietary supplements, with vitamins (77.3%) being the most used, followed by minerals (54.4%) and herbs or extracts (50.3%) [49]. The most common reason for using food supplements was the improvement of physical condition and treatment of nutrient deficiencies for both men and women, followed by the treatment or prevention of pathological conditions, such as anemias and osteoporosis in the group of women (31.3%) or the increase of muscle mass (31.0%) and sports performance (26.8%) in the case of men. Other reasons, such as weight loss (mean value of 13.4%) and improving mental function (mean value of 12.5%) were also mentioned by the participants. A recent systematic review of publications surveying the consumption of supplements globally during the first two years of the COVID-19 pandemic showed that in Asian countries, the use of these products ranged from 25% to 63%, in Middle Eastern countries from 15% to 71%, and in Europe from 21% to 80% [50]. This study also considered the use of herbal products as a separate class, indicating that about 50–64% of the inquired population had resorted to PFS, mostly containing immune-boosting natural products/plants, such as ginger, garlic, honey, lemon, and cinnamon.

Overall, the literature suggests that PFS corresponds to a considerable part of the food supplements consumed in the U.S. and Europe. By including herbal ingredients in the formulations, besides being advertised as a “natural product”, the pharmacological effect associated with the plant’s use as traditional herbal medicine is frequently suggested by the producers and often expected by the consumers of the product [51]. To achieve the desired effect and meet consumers’ expectations, PFS can be adulterated by the addition of drugs with the corresponding pharmacological activity. In addition to PFS tainted with pharmaceuticals, another practice seems to be emerging, namely, the adulteration of PFS by the addition of synthetic molecules that have been described to exist naturally in plants but in low amounts [51].

According to different literature reviews and the information available in public databases, PFS marketed for sexual potency, weight loss, and bodybuilding are the most prone to suffer adulteration by the addition of pharmaceuticals or synthetic unauthorized substances. Nevertheless, data suggest that other types of PFS, whose popularity has been on the rise, are increasing targets of adulteration. Among those, PFS advertised for cognitive enhancement, as they contain nootropic plants as ingredients, have risen in prominence worldwide, and their possible adulteration has been highlighted by different studies [52]. The direct use of nootropic substances, such as the pharmaceutical stimulant Adderall, is known to occur by a variety of consumers, including students, the military, and night-shift workers, who seek increasing concentration and focus, performance enhancement, and fatigue reduction [53]. Among college students, studies have reported the illegal use of compounds such as modafinil and methylphenidate, which were developed to treat disorders such as attention deficit, narcolepsia, and/or dementia-like diseases, and even illegal compounds, such as cocaine and amphetamines [54]. The analyzed literature suggests that globally, there is a growing prevalence of smart drugs’ consumption among students, the main reason being the improvement in concentration, performance, time optimization, and increase in free time [54]. To achieve these outcomes while avoiding the consumption of such substances, a higher number of consumers are increasingly resorting to PFS advertised with nootropic activity. For example, among college students, popular supplements used with the intent of improving cognitive function include those containing plants described as nootropics.

Besides PFS used for cognitive enhancement, other supplements used for different purposes but also related to brain activity, such as mood or sleep improvement, are also increasingly available in the market. Altogether, the global market for brain health supplements was estimated at USD 8.2 billion in 2022 and is expected to grow to more than USD 15 billion by 2030 [53]. As far as the literature consulted, data on the consumption prevalence of supplements to improve mood or sleep are still scarce or non-existent. However, the U.S. National Poll on Healthy Aging (NPHA) survey, targeting a community of older adults that included 1065 community-dwelling adults (aged 65–80), showed that about 35% suffered from sleep-specific difficulties and resorted to the use of sleep improvement products, namely 21.9% use over the counter sleep products, 12.5% consume herbal-containing products, and 8.3% use prescription medication [55]. In addition, it was recently reported that in the U.S., the use of melatonin, a key regulator of the circadian rhythm purportedly involved in sleep disorders and frequently added as an ingredient in PFS, has increased from 0.4% in 1999–2000 to 2.1% in 2017–2018 [56]. Although in low numbers, different studies have already reported the presence of pharmaceutical drugs such as phenobarbital, diazepam, and alprazolam in PFS marketed for improving sleep [12,45].

To date, extant reviews have provided valuable insights into the adulteration of food supplements by the addition of pharmaceutical drugs by addressing different analytical techniques employed for detecting and identifying such fraudulent practices. However, the predominant focus has been confined to specific classes of dietary supplements, particularly those most prone to be tainted with synthetic pharmaceutical adulterants, encompassing the categories of supplements intended for enhancing sexual potency [57,58], promoting bodybuilding or sports performance [59,60,61], or a combination thereof, addressing aspects such as weight loss, muscle development/sports performance, and sexual performance [11,62,63]. Additionally, some studies encompassed multiple supplement groups associated with conditions such as erectile dysfunction, obesity/overweight, diabetes mellitus, and hypertension [64]. However, according to the reviewed literature, there is a lack of comprehensive reviews specifically addressing supplements that are used for purposes other than those mentioned.

Therefore, this review aims to systematically address the existing knowledge on the adulteration of food supplements tailored for brain health, such as those used for cognitive, mood, and sleep enhancement, by providing a critical overview of the works carried out on the detection and identification of pharmaceutical drugs and unauthorized substances within this category of food supplements.

## 2. Analytical Approaches for Detecting Drug Adulteration in Brain Health PFS

Mood disorders, depression, anxiety, and stress are health problems that nowadays increasingly affect society in developed countries. Such disorders are often difficult to diagnose and/or are underestimated but are thought to affect millions of individuals worldwide, therefore negatively impacting the economy by decreasing the society’s active workforce [65,66]. Traditional treatments for clinical depression typically involve the use of antidepressants, which are medications also prescribed for a range of other diseases, including obsessive-compulsive disorder (OCD), generalized anxiety disorder, and post-traumatic stress disorder (PTSD) [65,67]. Although several antidepressant drugs belonging to different chemical families and subclasses are available, these can exhibit adverse effects, such as addiction, weight gain, nausea, insomnia, drowsiness, sexual dysfunction [68], and in some cases even an increased risk of suicidal behavior [13]. Considering these severe side effects, many patients have turned to other approaches, such as those based on PFS containing vitamins, minerals, amino acids, and botanical extracts [66]. Although not much is known regarding the extent of fraud in the class of PFS for mood improvement, antidepressant drugs such as fluoxetine have already been identified in other types of PFS, such as those used to improve sexual performance and to promote weight loss [69,70].

Another common health problem associated with contemporary societies regards insomnia and sleep disorders, estimated to regularly affect 9% of the population and 30% occasionally [71]. Sleep disorders can strongly impact society by increasing the risk of accidents due to sleep deprivation and by decreasing life quality and memory, while increasing the risk of dementia [72]. To deal with this problem, most frequently, medications such as sedatives and hypnotic drugs are prescribed, but they can have various side effects that limit the long-term use of these drugs [71,72]. As an alternative, several consumers resort to the use of medicinal plants or PFS, with an increasing number of products being available in the market to induce or improve sleep, including botanical ingredients such as valerian root (*Valeriana officinalis*), sour jujube seeds (*Ziziphus jujuba*), and kava (*Piper methysticum*) [72,73]. However, studies have reported the identification of different pharmaceutical adulterants in this type of PFS, mainly belonging to sedative-hypnotics and anxiolytics classes, such as barbiturates and benzodiazepines [12,45].

As mentioned in the previous section, PFS used to improve cognition are also rising in consumption and popularity, not only among specific groups, such as students who feel the pressure to perform well in exams and professionals who wish to stand out in their workplaces, but also among the elderly, who frequently exhibit memory loss due to aging processes. To this aim, a wide range of PFS, advertised as having nootropic activity for containing botanicals, such as *Panax ginseng*, *Ginkgo biloba*, *Centella asiatica*, *Withania somnifera*, *Bacopa monnieri*, *Paullinia cupana*, *Eleutherococcus senticosus*, *Rhodiola rosea*, *Schisandra chinensis*, and *Lepidium meyenii*, are now available in the market. Caffeine is the most known and widely used nootropic, but other substances, either from natural sources or synthetic, that can interfere with brain activity are being detected in PFS [42,43,52].

Generally, the search for adulterant compounds added to PFS relies on the use of separation methods coupled with advanced detection techniques for compounds’ identification and possibly their quantification. Undoubtedly, mass spectrometry (MS) stands out as the most suitable detection technique for this purpose due to its capability to provide high specificity and sensitivity [74,75]. Currently, several different MS systems are available, including those that provide high-resolution (HRMS) mass measurements, allowing for compounds’ accurate identification or structural elucidation. In the last years, besides single and triple quadrupoles (Q), ion trap, orbitrap, and time-of-flight (TOF) analyzers, hybrid systems that combine features of different mass analyzers for enhanced performance, such as Q-TOF and Q-Orbitrap, have been increasingly used for detecting tainted PFS. In addition, the versatility of MS has extended to high-throughput techniques, such as ambient mass spectrometry (AMS) and flow injection mass spectrometry (FIMS), which allow for direct analysis of samples with minimal preparation. Nevertheless, in what concerns specifically PFS, generally, MS is coupled with different separation techniques, mostly liquid chromatography (LC), offering an increased selectivity. The use of gas chromatography is not as common for identifying pharmaceutical adulterants in PFS because API molecules can thermally degrade, as described by Vanhee et al. [44]. Because PFS are highly complex matrices due to several botanical ingredients rich in a diversity of molecules, hyphenation with LC allows for the separation of compounds, thus adding an additional layer of selectivity while minimizing possible matrix effects that interfere with the MS signal of the analytes [63]. Table 1 provides the resumed information about the different studies conducted on the detection of illegally added pharmaceutical drugs to PFS marketed for their activity at the brain level. In this table, one can verify the predominance of MS detection hyphenated with a previous LC separation step, as it offers a more comprehensive and effective approach for the detection and identification of possible fraud.

Currently, LC–MS is being used as an advanced analytical platform to carry out either targeted or untargeted analysis [10]. The decision to opt for a targeted or untargeted approach mainly depends on the knowledge about the sample and its potential adulterants, ensuring alignment with the specific analytical objectives. Targeted analysis is usually the technique of choice when selected drugs are being searched, while untargeted analysis offers a more comprehensive and exploratory approach, suitable to search for new and emerging adulterants, including unknown compounds, such as analogues.

### 2.1. Targeted Analysis

When searching for the presence of predetermined adulterant pharmaceuticals in PFS, optimizing compounds’ extraction can be highly relevant. To this purpose, sample cleaning steps can assist in eliminating potential interfering compounds, thereby enhancing both selectivity and sensitivity. This aspect gains further relevance when employing less accurate and sensitive detectors, such as the diode array detector (DAD). Addressing this need, Kim et al. [88] tested various solvents and clean-up procedures, including different QuEChERS sorbents and PTFE syringe filters, for the extraction of 11 nootropic substances from liquid (oils and extracts) and solid (tablets, hard capsules, and powders) supplements, intended for analysis by UHPLC-DAD.

In some of the works conducted on PFS advertised for their activity at the brain level, specific compounds have been specifically targeted due to products openly declaring their inclusion, despite being unapproved drugs in the U.S. One notable example regards the detection of piracetam in dietary supplements sold in the U.S. While piracetam is a prescription drug in Brazil and some EU countries used for cognitive- and memory-related issues, it is not regulated or approved by the FDA. In 2018, Cohen et al. [43] identified five brands of dietary supplements available for online purchase containing piracetam as an ingredient. The authors acquired two samples of each product at different times, which were analyzed by LC-Q-TOF and quantified using the corresponding reference standard and hydromorphone-d6 as an internal standard, allowing the confirmation and quantification of piracetam in eight out of ten products. Later, the same authors acquired online several dietary supplements labeled as containing centrophenoxine (also known as meclofenoxate), a cholinergic drug prescribed in China and other countries for dementia, brain trauma, and other conditions, but also not approved in the U.S. [89]. In this study, the authors relied on an ultra-high-performance liquid chromatography photodiode-array (UHPLC-DAD) methodology to analyze the commercial samples, with centrophenoxine being detected at 225 nm and the identity of the compound being assigned based on the retention time and UV spectra of the corresponding reference standard. Previously, the same authors also validated a UHPLC-DAD methodology for the quantification of vinpocetine and picamilon, other unapproved nootropic drugs, which was applied to the analysis of supplements sold online as containing vinpocetine (*n* = 23) and picamilon (*n* = 31). The method allowed detecting 16 and 30 products containing vinpocetine and picamilon, respectively. More recently, UHPLC-DAD was also proposed to identify 11 nootropic substances in PFS advertised for brain health, memory, and cognition [88]. After method validation, it was applied to 55 commercial samples, allowing the detection of vinpocetine and kavain in 5 samples, which were further confirmed by comparing their MS spectra and fragmentation patterns with those of reference standards employing LC-Q-TOF/MS. Despite some works being developed based on DAD detection, as can be observed in Table 1, most elect MS detection over DAD, owing to its superior accuracy and sensitivity.

A similar strategy of selecting and acquiring supplements that declare on their labels to contain the substance under study was followed by Crawford et al. [52]. Nevertheless, in this case, the study concerned huperzine A, a substance that was recognized as a new dietary ingredient in 1997 [90], although not authorized in other regions, such as in the EU. A total of 22 supplements with claims to enhance cognitive performance and declaring huperzine A as an ingredient were selected and analyzed by LC-Q-TOF. Of these samples, four products listed “huperzine A” as an ingredient (with no claim for the plant material), six listed “*Huperzia serrata* extract (standardized to 1% huperzine A)”, and others listed aerial plant parts or extract of *Huperzia serrata*. In this study, a double approach was followed; first, the samples were qualitatively analyzed by LC-QToF-MS both in positive and negative modes, allowing the identification of compounds by accurate mass. In addition, an approach targeting huperzine A using a validated UPLC-DAD-QToF-MS/MS method operating in the positive mode allowed the compound’s identification by its MS/MS pattern and its quantification based on a reference standard. The targeted analysis revealed significant discrepancies between the label and actual composition. Of the 13 products that indicated the amount of huperzine A per serving size, only 2 were within 10% of the declared amount. In four supplements, two of which claimed an amount of 200 μg/serving size, the compound was found to be under the limit of quantification. Moreover, for several samples, the quantified amount of the compound was not in agreement with the inclusion of plant parts or extracts as ingredients. While seven supplements containing the plant extract also showed other compounds characteristic of the plant, another eight that included aerial parts or extract as ingredients showed the presence of huperzine A only. Additionally, the untargeted approach revealed that 16 supplements (73%) contained undisclosed ingredients, including some amino acids, different acids that can act as preservatives or flavoring agents, and more importantly, disclosed the presence of synthetic compounds, such as demelverine, 1,5-dimethylhexylamine (DMHA), and stimulants on the FDA advisory list, such as hordenine.

**Figure 1:**
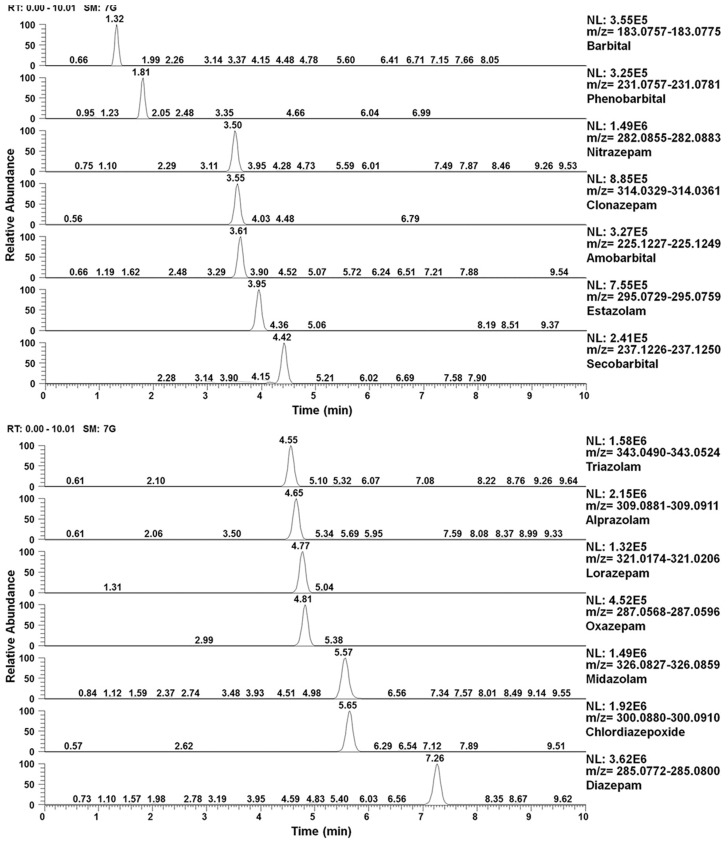
Extracted ion chromatograms in matrices spiked with 10 ng/g of sedative-hypnotics. Reprinted from [12] with permission from Taylor and Francis (license number 5747140088630).

Other studies have targeted specific groups of molecules likely to be added to certain types of food supplements, selected on the basis of their pharmacological activity aligning with the advertised effects of the supplement. Jiang et al. [12] developed and validated a UHPLC-Q-Orbitrap HRMS for the targeted detection of 14 barbiturates and benzodiazepines in supplements for sleep improvement. The analysis was performed in full MS/dd-MS^2^ mode, thus combining a simultaneous non-targeted, full-scan MS screening with a data-dependent (dd-) MS/MS acquisition for the 14 targeted compounds. The application of a defined isolation window at the quadrupole MS and the use of a specific inclusion list with monoisotopic masses and normalized collision energies for each of the precursors of the targeted molecules provided a high sensitivity while, at the same time, allowing for the untargeted screening of other possible adulterants in the sample. The authors validated the proposed method for detecting and quantifying the 14 selected sedative-hypnotics considering different parameters, such as selectivity, sensitivity, linearity, accuracy and precision, recovery, matrix effect, and stability. Figure 1 shows the extracted ion chromatograms of the 14 compounds in spiked matrices. The validated method was applied to the evaluation of 45 batches of dietary supplements, showing that 3 were adulterated with diazepam, clonazepam, and alprazolam at high levels [12]. A similar approach based in the use of LC-Q-Orbitrap HRMS operating in MS/dd-MS^2^ was proposed by Lee et al. [16] for the target analysis of 32 compounds, including 11 benzodiazepines, 13 synthetic cannabinoids, 5 amphetamines, and 3 benzylpiperazines. After validation, the method was applied to analyze 21 products advertised for weight loss, sleep induction, and concentration enhancement acquired from 2016 to 2018. The analysis of retention time, mass accuracy with a tolerance of 5 ppm, and MS^2^ fragments allowed to identify four benzodiazepines, namely, alprazolam, diazepam, estazolam, and lorazepam, and two synthetic cannabinoids in the samples. Of the 21 samples analyzed, 10 were illegally added with 1 benzodiazepine and only 6 were not adulterated. Lee et al. [45] suggested a slightly different approach for the analysis of 22 compounds, including barbiturates, benzodiazepines, zolpidem, and H1-antihistamines, since the authors first proposed screening the samples by using LC-Q-Orbitrap HRMS, with the positive ones being reinspected and quantified by a validated UHPLC-MS/MS method operating in the multiple-reaction monitoring (MRM) mode. A total of 144 samples advertised as sleep inducers for their natural ingredients were bought from 2014 to 2016, of which 2 were found to contain phenobarbital at high levels (>24.5 mg/g).

Shin et al. [77] opted for a multi-class LC-MS/MS method targeting 64 pharmaceutical drugs utilized for various therapeutic purposes. Depending on their function, these targeted compounds might be found in supplements designed for purposes ranging from sexual enhancement and weight loss to muscular strengthening and relaxation products. Among the targeted compounds, the validated method allowed the identification and quantification of 12 compounds with activity at the brain level, thus potential adulterants of the evaluated relaxation products. Those included the antidepressant fluoxetine, nootropics with potential to enhance cognitive function, such as vinpocetine and noopept, levodopa (a medication used in Parkinson’s disease), drugs or natural compounds with potential anxiolytic effects for their ability to increase GABA levels in the brain (picamilon and kavain), stimulants (amphetamine and beta-methylphenylethylamine), magnoflorine (a natural alkaloid that may have neuroprotective effects), and compounds structurally similar to melatonin and serotonin that may interfere with the sleep–wake cycle and mood regulation, such as 5-methoxytriptamine, 5-HTP, and melatonin. Of the 11 relaxation products evaluated, 1 contained kavain (18.8 mg/g), 1 melatonin (6.74 mg/g), and 4 were found to contain 5-HTP (56.6–226 mg/g). Kavain, magnoflorine, and picamilon were also analyzed by HPLC-MS/MS in dietary supplements, together with asarone (a natural compound found in *Acorus calamus*, traditionally used as a herbal remedy for anxiety and insomnia, and studied for its neuroprotective effects [91]) and other seven drugs for weight loss or sexual enhancement [77]. The validated method was applied to monitor 115 samples, resulting in the detection of kavain and magnoflorine in 2 samples.

Several other studies in the literature report the use of targeted LC-MS/MS for the quantification of drugs with potential effects at the brain level. However, the analyzed supplements purported activities other than brain-related functions, such as weight loss. Lee et al. [79] proposed the use of LC-Q-Orbitrap-MS to screen the presence of drugs in weight-loss supplements, followed by subsequent analysis of the identified adulterated products by LC-MS/MS, which was validated to allow the quantification of 45 compounds. In addition to banned anorexics, laxatives, and related compounds, weight-loss supplements have been found to contain substances with diverse pharmacological activities, including antidepressants and stimulants [11]. Therefore, in this work, the authors included several antidepressants (bupropion, sertraline, fluoxetine, and paroxetine), a stimulant and mood enhancer (2-phenethylamine), a sedative-hypnotic barbiturate (phenobarbital), and modafinil, a wakefulness-promoting medication. Among 656 products analyzed, 237 were found adulterated, with fluoxetine being detected in 9 samples. Roiffé et al. [80] developed and validated a targeted approach using a LC-Orbitrap-HRMS for the quantification of 111 illicit substances in whey protein food supplements. The substances belong to different classes and included different stimulants, such as methamphetamine, fenproporex, prolintane (a psychostimulant to increase alertness and focus), substances to treat ADHD and narcolepsy, such as pemoline, pipradol, and methylphenidate, psychoactive mood enhancers (methylenodioxyamfetamine and methylenodioxymethamphetamine), selegiline (monoamine oxidase inhibitor used to treat Parkinson’s disease and depression), and cognitive enhancers and wakefulness promoters (modafinil, pemoline, and cyclazodone).

One of the major difficulties when choosing a targeted approach is the need for reference standards, which are not always available. Vanhee et al. [44] well described this challenge when attempting to identify and quantify adrafinil in suspected illegal dietary supplements. Adrafinil is a prodrug that is converted to modafinil in the body, being classified as a psychostimulant and nootropic, and for which there was no reference standard available. To address this challenge, the authors employed a multi-methodological approach combining various analytical techniques, including GC-MS, LC-MS/MS, and nuclear magnetic resonance (NMR) spectroscopy. While the GC-MS revealed thermal degradation of the unknown molecule, the LC-MS/MS analysis confirmed the presence of ions consistent with adrafinil and its metabolites, supporting its presence in the samples. The identification of adrafinil and its quantitative estimation per capsule were finally achieved by employing NMR spectroscopy [44].

### 2.2. Untargeted Analysis

In this approach, the sample is screened by HRMS analysis, most frequently coupled with a previous chromatographic separation step. The acquired full HRMS data are then subjected to comprehensive data mining based on accurate masses and relative isotopic abundances to screen for unknown compounds in the sample [63]. Because untargeted analysis aims to provide a representative and comprehensive snapshot of the compounds present in the sample, the associated extraction procedure should be as broad and simple as possible to capture a wide range of analytes while avoiding undesirable loss of compounds [92,93]. By extracting a diverse array of compounds, untargeted approaches maximize the chances of detecting unknown or unexpected adulterants. In general, for liquid samples, a simple “dilute and shoot” procedure is advised, consisting only of the filtration of the sample, which may be diluted if needed, and its injection in the chromatographic system. For solid samples, mixtures of organic solvents (e.g., methanol, ethanol, and acetonitrile) with water are generally used, aiming for the recovery of compounds of different polarities, together with shaking, sonication, and/or vortexing to increase the extraction efficacy [10,57,63,94,95]. The extract is then filtered and injected or taken to dryness, redissolved in a known amount of an adequate solvent, and injected.

Besides allowing structure elucidation of compounds and the discovery of new and unexpected adulterants, such as analogues, untargeted analysis also has the advantage of enabling retrospective data analysis. This capability is particularly relevant when emerging adulterants are identified and there is an interest in re-analyzing old samples to search for their existence.

**Figure 2:**
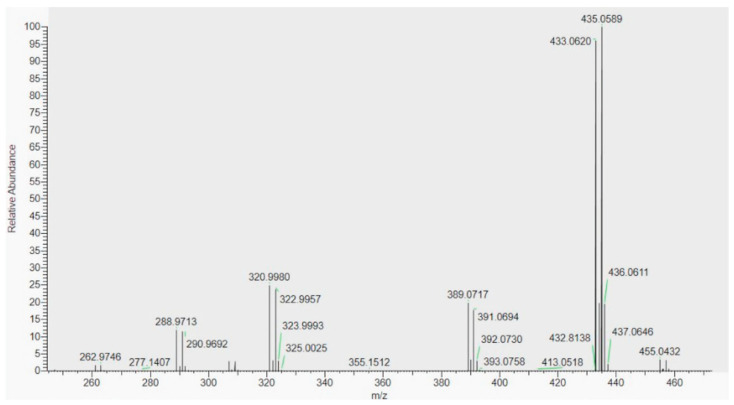
Full-scan mass spectrum of peak eluting at 3.9 min in the HR-LCMS Orbitrap analysis of the U-Dream product. Reprinted from [15] under the terms of the Creative Commons Attribution License.

The potential of elucidating compounds’ structures is pivotal in the identification of drug analogues and has allowed the detection of new substances added in PFS with activity at the brain level. The work of Brown et al. [15] is a very good example of such capabilities. In 2020, the authors examined products of the U-Dream line, marketed as a natural health product for sleep aids in Canada and as dietary supplements in the U.S., following diverse reports of side effects by numerous consumers. Based on the received information, a study was undertaken to evaluate the possible presence of undeclared APIs, with an initial focus on known prescription drugs used to treat insomnia. With this aim, first, the presence of lorazepam was evaluated by HPLC-ESI-Q-TOF. The analysis did not show the presence of this compound but an unknown peak showing a characteristic bromine isotopic pattern was evidenced. Therefore, on a second approach, full-scan positive ESI data were acquired using a Q-Exactive Orbitrap (Figure 2). By comparing the mass spectrum of the unknown with several halogen-containing compounds with sedative activity, the authors concluded that the obtained data were consistent with a brominated analogue of the pharmaceutical sedative zopiclone. This putative identification was further confirmed by ^1^H NMR analysis of the isolated compound and by the positive results obtained using a commercial zopiclone/eszopiclone enzyme-linked immunosorbent assay (ELISA) kit.

An untargeted approach was also used in the work of Cohen et al. [42] in the analysis of selected dietary supplements sold in the U.S. labeled as containing analogues of piracetam, namely, omberacetam (also known as noopept), aniracetam, oxiracetam, and phenylpiracetam. While some of these analogues are used as prescription drugs in countries other than the U.S., others have been studied for their capability in treating dementia and other neurological conditions, yet none were approved by the FDA. The use of such approach, instead of targeting just the four mentioned compounds, allowed the detection of three additional unapproved drugs (vinpocetine, phenibut, and picamilon), also considered to have activity at the brain level, as well as two out of the four analogues (omberacetam and aniracetam). The presence of the identified drugs was subsequently confirmed using reference standards, followed by their quantification. Omberacetam is not an approved drug neither in the U.S. nor the EU, yet it has been tested in human clinical trials using a pharmacological dose of 20 mg [96]. In one product, omberacetam was detected at a much higher quantity, namely at a dose of 40.6 mg. Curiously, the other two analogues despite being labeled in three products, were not detected.

## 3. Conclusions

In recent years, different groups of the population are increasingly concerned about healthy lifestyles as well as improving their cognitive function or maintaining brain health. Simultaneously, contemporary societies and modern professional life are contributing to increasing problems, such as stress, anxiety, depression, sleep disturbances, and memory loss. Moreover, there has been a higher incidence of neurodegenerative disorders together with the absence of effective treatments. All these aspects potentiate the consumption of plant food supplements tailored for maintaining/improving brain health due to containing plant material or extracts with well-recognized pharmacological activity at the central nervous system. This trend is particularly evident among students and the elderly, but also in adults in general that are increasingly turning to these products as natural alternatives to improve cognitive performance, memory, mood, motivation, and/or sleep quality. However, emerging reports also suggest a growing trend of adulteration within this category of PFS by the addition of pharmaceuticals, such as sedatives, hypnotics, and nootropics, among others, underscoring the need for advanced and accurate analytical methods to detect such fraud cases. Addressing this need, MS hyphenated with chromatographic methods has emerged as an indispensable tool in identifying adulterants in PFS. The evolution of MS-based techniques, especially with the possibility of high-resolution mass spectrometry (HRMS) achieved through hybrid detectors, holds promising prospects for enhancing detection sensitivity and specificity.

Targeted analysis, employing tandem MS, stands out for its ability to precisely detect and quantify known adulterants, offering rapid data treatment and accurate results. However, only predetermined compounds are actively sought for and it is limited by the necessity for reference standards for all potential adulterants, which may not always be readily available. In contrast, untargeted analysis enables the screening of a broad spectrum of drugs without prior knowledge of their presence, facilitating the detection of both known and novel adulterants, including analogues. This approach also has the advantage of allowing for retrospective data analysis and sample re-evaluation, if necessary, for example if information on a new adulterant drug emerges. Nonetheless, untargeted analysis presents challenges in handling large volumes of complex data, requiring specialized expertise for their handling and interpretation. Considering the distinct advantages and limitations of both targeted and untargeted approaches, recent studies have demonstrated their complementary use in screening, identifying, and quantifying drug adulterants in PFS targeting brain health.

In the future, it is anticipated that HRMS will be increasingly used for the preliminary screening of samples, particularly those suspected of containing adulterating substances, such as PFS obtained from patients hospitalized following their consumption. Additionally, the use of untargeted HRMS-based approaches is expected to rise, aiming to detect potential new analogues clandestinely developed and employed as PFS adulterants.

As evidenced in this review, leveraging the strengths of each technique contributes to a comprehensive strategy for safeguarding consumer safety and product quality in the rapidly evolving landscape of plant food supplements’ adulteration.

## Figures and Tables

**Table 1:** Application of mass spectrometry approaches to detect drugs with activity at the brain level illegally added to food supplements.

PFS	Analytes ^a^	Sample Preparation	Separation Conditions (Column/Mobile Phase/Elution)	Run Time	MS System	Ionization/Detection Mode	Quantitative	Adulterated/Total Samples	Ref.
Nootropic/cognitive enhancing	Piracetam	Powder from capsules extracted with methanol, vortexed, centrifuged, and supernatant serially diluted in 10% acetonitrile	Agilent Poroshell 120 C-18 column (2.1 × 100 mm, 2.7 µm), T = 55 °C, mobile phase: water with 0.05% formic acid and 5 mM ammonium formate (A) and acetonitrile with 0.05% formic acid (B), injection volume 2.5 µL	12 min	LC-Q-TOF/MS	ESI (+)	Yes	2/10 (10 products of 5 brands)	[43]
Nootropic/cognitive enhancing	Omberacetam (noopept), aniracetam, phenylpiracetam, oxiracetam, phenibut, vinpocetine, picamilon	Powdered material sonicated in methanol, centrifuged, and filtered (0.45 µm PTFE membrane). Finally, samples were further diluted ×5, ×10, and ×100, vortexed, and sonicated.	Agilent Poroshell 120 EC-C18 (2.1 × 150 mm, 2.7 µm), T = 35 °C, mobile phase: water with 0.1% formic acid (A) and acetonitrile with 0.1% formic acid (B), flow rate 0.2 mL/min, injection volume 2 µL	38 min	LC-QTof-MS	ESI (+)	Yes	10/10	[42]
Nootropic/cognitive enhancing	Adrafinil	Sample is solubilized in methanol, sonicated, and filtered (0.2 μm PTFE)	GC: VF-5 ms column (30 m × 0.25 mm × 0.25 μm film thickness)	55 min	GC-EI-Q	EI	Yes (by NMR)	1/1	[44]
			LC: ACQUITY UPLC BEH C18 Column (150 × 2.1 mm, 1.7 μm particle size), mobile phase: 0.1% formic acid in water (A) and 0.1% formic acid in acetonitrile (B), T = 45 °C; flow rate was 0.5 mL/min	13 min	LC-ESI-IT-MS/MS	ESI (+)			
Nootropic/cognitive enhancing	Huperzine A, demelverine, 1,5-dimethylhexylamine, 1,3-dimethylhexylamine, N-phenethyldimethylamine, halostachine, higenamine, noopept, phenylethylamine, vinpocetine, and sulbutiamine	Powder was solubilized in 10% hydrochloride, added with methanol, sonicated (30 min), centrifuged, supernatants were combined, volume adjusted to 10 mL, and filtered (0.45 µm PTFE)	Qualitative: C18 column, T = 35 °C, mobile phase: water and acetonitrile, both with 0.1% formic acid, flow rate 0.2 mL/min using a gradient elution.	19.5 min	LC-Q-TOF-MS	ESI (+/−)	Yes	16/22	[52]
			Quantitative: Waters ACQUITY UPLC HSS T3 column (100 × 2.1 mm i.d., 1.8 μm), mobile phase: 0.05% formic acid in water (A) and acetonitrile containing 0.05% formic acid (B), flow rate of 0.4 mL/min.		UPLC-DAD-Q-TOF	ESI (+)			
Sleep aid	14 sedative/hypnotics (4 barbiturates and 10 benzodiazepines)	Sample extracted with methanol, vortexed, ultrasonic treatment (15 min), centrifuged, and supernatant filtrated (0.22 μm membrane)	Hypersil GOLD aQ C_18_ column (100 × 2.1 mm, 1.9 μm), T = 30 °C, mobile phase: water (A) and acetonitrile (B), both containing 0.1% formic acid, flow rate 0.3 mL/min	10 min	UHPLC-Q-Orbitrap-MS	HESI (+/−)	Yes	3/45 (adulterated with diazepam, clonazepam, and alprazolam)	[12]
Sleep aid	Lorazepam, lorazepam glucuronide, zopiclone analogue	Powder extracted with acetonitrile:water (1:1) or with MeOH:water (4:1) in the targeted and untargeted approaches, respectively; sonicated or vortexed; centrifuged and filtrated	Targeted analysis: Agilent Poroshell 120 SB-C18 (2.7 μm, 2.1 mm × 100 mm column), T = 30 °C, mobile phase: water:acetonitrile, flow rate 0.5 mL/min	17 min	HPLC-ESI-QTOF (targeted)	ESI (+)	No	1/1 (U-Dream product adulterated with zopiclone analogue)	[15]
			Untargeted analysis: Kinetix Polar C18 (2.6 μm, 100 × 3.0 mm column), T = 40 °C, mobile phase: 0.1% formic acid in water (A) and 0.1% formic acid in MeOH (B), flow rate 1.6 mL/min, injection volume 10 μL		UPLC-DAD/Q- Orbitrap (untargeted)	ESI (+)			
Sleep aid	22 sedative-hypnotics (12 benzodiazepines, 6 antihistamines, 3 barbiturates and zolpidem)	Sample extracted with 70% MeOH, sonicated (30 min), filtered (0.2 μm PTFE), and diluted with methanol	Qualitative: BEH-C18 column (100 × 2.1 mm ID, 1.7 μm), T = 30 °C, mobile phase: 0.1% formic acid in both deionized water (A) and acetonitrile (B), flow rate 0.25 mL/min, injection volume 1 µL	13 min	Q-Orbitrap-MS	HESI or ESI (+) except for phenobarbital and pentobarbital (−)	Yes	2/46 food supplements (adulterated with phenobarbital)	[45]
			Quantitative: Waters ACQUITY UPLC BEH C18 column (2.1 × 100 mm, 1.7 μm, Waters), T = 30 °C, mobile phase: 0.1% formic in deionized water (A) and acetonitrile (B), flow rate 0.25 mL/min, injection volume 1 μL		UHPLC-QQQ-MS/MS				
Sleep aid	32 compounds (11 benzodiazepines, 13 synthetic cannabinoids, 5 amphetamines, and 3 benzylpiperazines)	Sample extracted with methanol, sonicated (30 min), volume adjusted, and filtered (0.2 μm PTFE)	BEH C18 column (100 × 2.1 mm ID, 1.7 μm), T = 35 °C, mobile phase: 0.1% formic acid in both deionized water (A) and acetonitrile (B), flow rate 0.25 mL/min, injection volume 1 μL	15 min	LC-Q-Orbitrap	HESI (+)	Yes	15/21 (alprazolam, diazepam, estazolam, lorazepam and two synthethic cannabinoids were detected)	[16]
To alleviate depression, anxiety, and insomnia	22 antidepressants, anxiolytics, and ADHD medication	Samples mixed with methanol 70%, sonicated, mixed, volume adjusted to 50 mL, and filtered (0.22 μm PTFE)	Eclipse Plus C18 column (2.1 × 100 mm, i.d. 1.8 μm), T = 40 °C, mobile phase: DW containing 0.1% formic acid (A) and methanol containing 0.1% formic acid (B), flow rate was 0.2 mL/min, injection volume 2.0 μL	13 min	LC-Q-Orbitrap-MS	HESI (+)	Yes	2/118 (fluoxetine detected)	[76]
			Poroshell 120 EC-C18 column (2.1 × 100 mm, 2.7 μm), T = 40 °C, mobile phase: distilled water containing 0.1% formic acid (A) and methanol containing 0.1% formic acid (B), flow rate 0.3 mL/min injection, volume 2.0 μL	17 min	HPLC-Q-Trap	ESI (+)			
Relaxing and others (sexual enhancement, weight loss, muscle building)	64 compounds, including stimulants, antidepressants, levodopa, nootropics, serotonin, melatonin, mexamine, 5-HTP	Samples were mixed in 15 mL water, added with 20 mL methanol, sonicated (20 min), volume adjusted to 50 mL, and filtered (0.22 µm PTFE)	ACQUITY BEH C18 column (2.1 mm × 150 mm, 3.5 µm), T = 40 °C, mobile phase: A (0.1% formic acid in water) and B (0.1% formic acid in acetonitrile), flow rate 0.3 mL/min, injection volume 5 µL	25 min	UPLC-QQQ-MS/MS	45 compounds ESI (+), 19 ESI (−)	Yes	6/11 relaxing products (kavain, melatonin, 5-HTP detected)	[77]
Relaxing and others (sexual enhancement, weight loss)	11 compounds, including asarone, kavain, magnoflorine, and picamilon	Samples were mixed in 15 mL water, added with 20 mL methanol, sonicated (20 min), volume adjusted to 50 mL, and filtered (0.22 µm PTFE)	ACQUITY BEH C18 column (2.1 mm × 150 mm, 3.5 µm), T = 40 °C, mobile phase: A (0.1% formic acid in water) and B (0.1% formic acid in acetonitrile), flow rate 0.3 mL/min, injection volume 5 µL	25 min	UPLC-QQQ-MS/MS	ESI (+)	Yes	3/115 (kavain, magniflorine, dihydroepiandrosterone)	[78]
Other (weight loss)	45 compounds, including antihistamines, stimulants, antidepressants, nootropic, others	Sample was dissolved in methanol solution and sonicated and filtered through a PTFE filter with a pore size of 0.2 µm	Qualitative and quantitative: BEH C18 column (100 × 2.1 mm, 1.7 μm), T = 30 °C, mobile phase: A (0.1% formic acid in 5% ACN) and B (0.1% formic acid in 95% ACN), flow rate was 0.25 mL/min, injection volume 2 μL	15 min	UHPLC-Q-Orbitrap-MS	HESI (+/−)	Yes	237/656 (mainly adulterated with anorexics, laxatives, and stimulants)	[79]
					UPLC-QQQ-MS/MS	ESI (+/−)			
Other (whey protein food supplements)	105 compounds, including stimulants, phenylpiracetam, modafinil, methylphenidate, pipradol, pemoline, prolintane, selegiline	Sample extracted with water, vortexed, and centrifuged. One aliquot was added with acetic acid solution, another submitted to SPE, and the eluted solution evaporated under N_2_ flow. The first aliquot was added to the residue and homogenized	Zorbax SB-C18 column (3.0 mm × 50 mm, 1.8 μm), T = 40 °C, mobile phases: 0.1% ammonium formate/0.1% formic acid in water (A) and 0.1% formic acid in methanol (B). The flow rate was 600 μL min^−1^ and the injection volume was 0.5 μL	14 min	LC-Q-Orbitrap-MS	ESI (+/−)	Yes	7/11 whey protein food supplements	[80]
Others (weight loss, thermogenicals, meal replacement)	32 compounds, including anxiolytics, antidepressants, and stimulants	Dilution in MeOH, sonicated, dilution in 0.05% formic acid in water/acetonitrile the (mobile phase), filtration through a Teflon membrane (0.2 μm)	UHPLC Zorbax model SB-C_18_ (Agilent^®^) column (2.1 × 50 mm, 1.8 μm); gradient elution program with 0.05% formic acid in water/acetonitrile as mobile phases, flow rate of 0.6 mL min^−1^, T = 50 °C	19 min	UHPLC-QQQ-MS/MS	ESI (+/−)	Yes	80/108 (adulterated with caffeine or synephrine)	[4]
Others (sexual enhancement, weight loss, muscular strengthening)	80 compounds, including stimulants and fluoxetine	Sample mixed with water, then methanol was added, sonicated, and supernatant filtered (0.22 μm PTFE)	LC-MS/MS—ACQUITY UPLC^®^ HSS C18 column (2.1 × 150 mm, 1.8 μm), T = 40 °C, mobile phase: 0.1% (*v*/*v*) formic acid in water (A) and 0.1% (*v*/*v*) formic acid in acetonitrile (B), flow rate was 0.3 mL/min, injection volume was 5 μL	25 min	UPLC-MS/MS—QQQ	ESI (+/−)	Yes	51/51	[81]
Other (pain relief, diabetes, weight increase, weight loss)	18 compounds, including stimulants and diazepam	Sample extracted with MeOH, stirred, sonicated, diluted with the mobile phase, and filtered (0.4 μm Millipore membrane)	Poroshell 120 EC C18 column (3.0 ID × 100 mm length, 2.7 μm), T = 40 °C, mobile phase: 0.1% formic acid in water (A) and 1% formic acid in 15% ACN and 85% methanol (B), flow rate 0.3 mL/min, injection volume 1 μL	27 min	LC-QQQ-MS/MS	ESI (+/−)	Yes	7/33	[82]
Not referred (soft-gel-type dietary supplements)	92 compounds, including 4 antidepressants, 1 hypnotic-antidepressant, 2 anxiolytics, 1 nootropic, 1 medication for ADHD	Analytes were extracted from samples by three protocols, QuEChERS-dSPE, EMR-lipid dSPE, and dispersive liquid–liquid microextraction (DLLME), for evaluation of sample clean-up	Waters ACQUITY^®^ UPLC BEH C18 column (150 × 2.1 mm, ID, 1.7 µm), T = 40 °C, mobile phase: 0.1% (*v*/*v*) formic acid in water (A) and acetonitrile (B), flow rate 300 µL/min, injection volume 2 µL	15 min	UHPLC-Q-TOF-MS	ESI (+)	Yes	0/10	[83]
Not referred (dietary supplements)	fluoxetine	Samples mixed with methanol, added with an alkaline buffer solution and tertiary butyl methyl ether, and centrifuged. Liquid nitrogen was used to freeze the bottom non-organic layer, then evaporate; samples were reconstituted with mobile phase, vortexed, and centrifuged	Phenomenex Gemini C18 NX column (50 × 2 mm, 5 μm), T = 35 °C, mobile phase: acetonitrile: 0.1% formic acid (1:1, *v*/*v*), flow rate 0.5 mL/min, injection volume 20 µL	~5 min	HPLC-QQQ-MS/MS	-	Yes	75/138	[84]
Supplements to improve wellness, including mental conditions, sexual performance, sports performance, and weight loss	124 compounds, including 8 stimulants, 12 anxiolytic/hypnotic benzodiazepines, 5 antipsychotics/neuroleptics, 1 antihistamine with sedative properties, 2 sedatives	Samples were extracted two times with methanol/water, vortex-mixed, sonicated, centrifuged, and supernatants filtered through C18 SPE cartridges pre-conditioned with methanol	Kinetex XB-C18 column (3.0 mm × 100 mm, 2.5 μm), with a guard column Phenomenex C18 (40 mm × 2.1 mm), T = 40 °C, mobile phase: formic acid 0.1% (A1)/methanol (B1) and acetic acid 0.1% (A2)/acetonitrile (B2) for the positive and negative acquisition, respectively, flow rate 0.45 mL/min, injection volume 10 μL	20 min	LC-Q-Orbitrap-MS	HESI II (+/−)	Yes	5/110	[85]
Several types ^b^	A database with over 1500 compounds was used	Samples shaken 30 min with water/acetonitrile (20/80) containing 1% acetic acid, centrifuged, 250 μL transferred to a filter vial, diluted with water, and filtered (if necessary, further diluted with MeOH/water or ACN/water)	Untargeted (full-scan HRMS): Atlantis T3 LC column (100 × 3 mm, 3 μm), T = 40 °C, mobile phases: water (A) and methanol/water 95:5 (*v*/*v*) (B), both containing 2 mM ammonium formate and formic acid, flow rate 300 μL/min, injection volume 5 μL	20 min	UPLC-Q-Orbitrap-MS	HESI II (+/)	Yes	264/416 (among others, phenethylamine, DMAA, DMBA, ephedrine, octopamine, β-methylphenethylamine, fluoxetine, kavain, DMAE)	[86]
			Targeted (quantitative): Atlantis T3 column (100 × 3 mm, 3 μm), T = 30 °C, mobile phases: water (A) and methanol/water 95:5 (B) both containing 5 mM ammonium formate and 0.1% formic acid, flow rate 400 μL/min, injection volume 5 μL	21 min	LC-QTRAP-MS/MS	ESI (+)			
Melatonin-containing food supplements	Serotonin (melatonin determined by electrochemical detection)	Samples extracted with methanol, filtered (0.45 μm, EMD Millipore), and diluted in Milli-Q water. Liquid samples were directly diluted and injected	Waters ACQUITY UPLC BEH C18 column (2.1 × 50 mm, 1.7 µm), T = 40 °C, mobile phase: 10 mM ammonium acetate, adjusted to pH 9 with ammonium hydroxide (A) and acetonitrile (B), flow rate 0.5 mL/min, injection volume 7.5 µL	~5 min	LC-QDa	-	Yes	15/30 (difference of melatonin > 15% compared to label) 8/30 (serotonin detected)	[87]

^a^ When multiple compounds are detected, focus is given to compounds that act at the brain level, with the other classes not being mentioned. ^b^ Samples collected by the Netherlands Food and Consumer Product Safety Authority (NVWA) either after a RASFF alert or a consumer health complaint, or in proactive analysis of products with emphasis on PFS for sexual improvement, pre-workout, and weight loss. MeOH: methanol; ACN: acetonitrile.

## Data Availability

The original contributions presented in the study are included in the article, further inquiries can be directed to the corresponding authors.
